# Analysis of the impact of the learning curve and other factors on the duration of TaTME procedures

**DOI:** 10.1007/s10151-026-03299-4

**Published:** 2026-03-31

**Authors:** B. Kapturkiewicz, M. Kazanowski, P. Lesiak, D. Ramsey, M. Bebenek

**Affiliations:** 1 1st Department of Surgical Oncology, Lower Silesian Oncology, Pulmonology and Hematology Center, Pl. Hirszfelda 12, 53-413 Wroclaw, Poland; 2 Department of Oncology and Haematology, Faculty of Medicine, Wroclaw University of Science and Technology, Wroclaw, Poland; 3 Department of Computer Science and Systems Engineering, Wroclaw University of Science and Technology, Wroclaw, Poland

**Keywords:** TaTME, Learning curve, Operative time, Rectal cancer, Surgical training

## Abstract

**Background:**

Transanal total mesorectal excision (TaTME) is a technically demanding procedure with a significant learning curve. Operative time is often used as a proxy for procedural proficiency.

**Objective:**

To evaluate the impact of the learning curve and selected patient- and procedure-related factors on the operative duration of TaTME procedures.

**Methods:**

A retrospective analysis was conducted on 250 consecutive patients who underwent TaTME for rectal cancer between 2016 and 2025. Operative time trends were assessed in relation to case sequence, individual surgeon experience, and patient-related variables including sex, body mass index (BMI), ASA score, and technical aspects such as protective ileostomy creation.

**Results:**

The mean operative time across the cohort was 200.52 min. A significant reduction in operative duration was observed over time, with stabilizing after approximately 100 cases. Male sex, higher BMI, and ASA scores ≥ 3 were independently associated with longer operative times. The use of a protective ileostomy also significantly increased operative duration. After adjusting for surgeon experience and case complexity, no difference remained between laparoscopic and open approaches. Substantial interoperator variability was observed, particularly in early cases.

**Conclusions:**

TaTME is associated with a steep learning curve, which significantly increases operative time. Patient-related factors such as sex, BMI, and comorbidities further influence surgical duration. These findings support the need for structured training pathways, outcome monitoring, and individualized preoperative planning.

## Introduction

### Background

Transanal total mesorectal excision (TaTME) is a surgical technique that has introduced new possibilities in the treatment of patients with low rectal tumors. First described in 2010, the procedure combines a transanal approach with laparoscopic abdominal access and serves as an alternative to classical total mesorectal excision (TME) performed via open or laparoscopic techniques [1, 2]. TaTME enables more precise and safer dissection in the lower pelvis, particularly in men, obese patients, and individuals with challenging pelvic anatomy. This may translate into improved oncological and functional outcomes [3].

However, implementing this technique poses significant organizational and technical challenges. Literature emphasizes that TaTME is associated with a steep learning curve, and achieving full proficiency by a surgical team may require performing more than 40 procedures [4]. In the early training phase, the duration of the surgery may be considerably prolonged, representing a burden for both the patient and the surgical team. Additionally, many other factors—such as patient BMI, sex, pelvic anatomy, tumor location, and the use of one-team or two-team approaches may influence the length of an operation [5, 6].

Understanding the influence of the learning curve and the aforementioned variables on the duration of surgery is crucial for optimizing resources, reducing anesthesia time, and minimizing the risk of intra- and postoperative complications. Moreover, it may contribute to the development of standardized protocols for TaTME implementation in new centers, including recommendations on the minimum experience required before performing the procedure independently. This is particularly relevant in the context of reports from Norway, where national registry data revealed alarmingly high local recurrence rates in TaTME cases performed during the early implementation phase. These findings led to a temporary nationwide moratorium on the use of TaTME [7]. This case highlights the necessity of carefully assessing a surgical team's readiness and continuously monitoring both oncological and technical outcomes throughout the adoption of TaTME.

### Study rationale

The aim of this study is to evaluate the impact of the learning curve and selected clinical and technical factors on the duration of TaTME procedures. The analysis seeks to identify variables significantly affecting procedure length and to assess the dynamics of changes in the length of operations as a surgical team gains experience.

### Aim of the study

1. To assess the impact of the learning curve on the duration of TaTME operations, including the identification of a turning point after which the length of procedures stabilizes
2. To analyze selected patient-related factors, including:
  - Body mass index (BMI)
  - Sex,
  - Location of the tumor within the rectum
  - Prior neoadjuvant treatment
3. To evaluate technical factors, such as:
  - Use of hybrid versus fully laparoscopic techniques
  - Presence or absence of a diverting ileostomy
4. To compare the length of operations in the initial versus advanced phase of implementing TaTME at the Lower Silesian Oncology, Pulmonology, and Hematology Center (LSOPHC)
5. To formulate recommendations regarding the minimum experience required by a surgical team before independently performing TaTME, with reference to evidence from the literature and cases of failure (e.g., the Norwegian report [7])

Achieving these objectives is aimed at improving our understanding of factors influencing procedural efficiency for TaTME operations and providing a foundation for recommendations on developing the training and credentialing of surgical teams.

## Materials and methods

### Study design and population

A retrospective analysis was conducted on patients who underwent transanal total mesorectal excision (TaTME) for low rectal cancer between May 2016 and February 2025 at the Lower Silesian Oncology, Pulmonology, and Hematology Center (LSOPHC). All procedures were performed with curative oncological intent. The indication for TaTME was the presence of a tumor in the distal rectum.

Patients were eligible for inclusion if they met the following criteria:

- Age ≥ 18 years
- Tumor located ≤ 6 cm from the anorectal junction (ARJ) as assessed by imaging, endoscopy, and digital rectal examination
- Underwent total mesorectal excision via the TaTME approach
- Availability of complete surgical data, including operative duration

Patients with incomplete or missing data were excluded from the analysis. In total, 250 TaTME operations were included in this study.

### Data collection and variables

- Clinical, anthropometric, and surgical data were obtained from medical records and a prospectively maintained institutional database. The following variables were collected and analyzed:
- Patient demographics (age, sex, body mass index [BMI])
- Tumor characteristics, including distance from the anal verge and receipt of neoadjuvant therapy
- Surgical details such as operative time (measured from skin incision to final closure), type of abdominal approach (laparoscopic, open, or conversion), and use of a diverting ileostomy
- Comorbidity indicators, including the American Society of Anesthesiologists (ASA) classification and World Health Organization (WHO) frailty score
- Identity of the primary operating surgeon and case sequence number, which allowed assessment of both team and individual learning curves

### Operative approach

All procedures were performed synchronously by two teams: a laparoscopic abdominal team and a transanal team. During the early phase of implementation, the abdominal phase was completed via an open approach because there were too few surgeons experienced in laparoscopy. Once the team expanded, the standard procedure became fully minimally invasive (laparoscopy combined with the transanal phase). Only sporadic open procedures were performed later in the series (≈10 cases), mainly for logistical reasons or exceptional complexity. This distribution of techniques (open vs laparoscopic vs conversion) was incorporated into the statistical models.

### Statistical analysis

Descriptive statistics were used to summarize patient demographics, clinical characteristics, and operative parameters. Continuous variables were expressed as means with standard deviations (SD) or medians with interquartile ranges (IQR), as appropriate, while categorical variables were presented as frequencies and percentages.

The relationships between operative time and patient- or procedure-related factors were initially assessed using univariate analyses, including Welch’s *t-*tests, analysis of variance (*ANOVA*), and Spearman’s correlation coefficients. Variables showing statistical significance (*p* < 0.05) in univariate analysis were subsequently entered into multivariate regression models.

Two multiple regression models were developed to identify independent predictors of operative duration. Both models included patient characteristics (age, sex, BMI, ASA classification, frailty status), technical factors (use of ileostomy, surgical approach), and indicators of experience (case sequence and operating surgeon). The selection of variables for the final models was based on stepwise elimination using the Akaike information criterion (AIC) to optimize model fit and parsimony.

Model performance was evaluated by comparing AIC values and examining residual plots for goodness of fit. All statistical analyses were performed with a two-tailed significance level set at *p* < 0.05.

## Results

A total of 250 TaTME procedures were analyzed. The results are presented below according to descriptive, univariate, and multivariate analyses, followed by a comparison of operative techniques and short-term oncological and clinical outcomes.

### Descriptive statistics and univariate analysis

A total of 250 TaTME procedures were analyzed. The results are presented below according to descriptive, univariate, and multivariate analyses. These operations were carried out by four different principal surgeons. Two of these surgeons led these operations from the beginning of the study period (nos. 1 and 3). The other two lead surgeons initially served as members of the surgical team and began leading operations just before the middle of the study period. Table 1 provides information about the number and mean length of operations carried out by each of the surgeons.

**Table 1:** Descriptive statistics regarding the operations led by the four principal surgeons

Surgeon	Mean length ± SD (min)	No. of operations	First operation (no.)
1	209.10 ± 48.4	89	1
2	204.55 ± 40.09	22	70
3	194.22 ± 43.93	103	3
4	194.86 ± 37.58	36	113

The mean length of operations was 200.52 (SD 44.73) min. Initial analysis indicated that the mean length of an operation was not associated with the primary surgeon (analysis of variance, *p* = 0.108). Importantly, in eight of the operations, there were specific clinical reasons for not performing an anastomosis (e.g., when using Hartmann’s procedure). These cases were excluded from further analysis (but were included when defining the team's and the principal surgeon's experience). After removing these cases, no association remained between the principal surgeon and the average operation length.

Of the corresponding 242 patients, 169 were male (69.83%) and 96 were assessed as being frail according to the WHO criteria (39.67%). The frequencies of the ASA risk categories among these patients are presented in Table 2.

**Table 2:** Distribution of the ASA category among patients

ASA category	1	2	3	4
Frequency	11 (4.55%)	193 (72.27%)	42 (17.36%)	2 (0.83%)

The patients were aged between 26 and 80 (mean 60.95, SD 10.41) years. The BMI ranged between 17.75 and 41.28 (mean 26.52; SD 3.91) kg/m^2^. Open surgery was used in 34 of the operations (14.05%), while a laparoscope was used in the remaining operations. Notably, open surgery was almost exclusively used at the beginning of the study period (the first 27 operations). Also, operations on patients categorized as frail were carried out on average later than operations on non-frail patients (Wilcoxon rank test, $$p = 0.00003).$$ This indicates that the surgical team progressively undertook more complex procedures.

There is a clear positive correlation between a patient's BMI and the length of an operation (Spearman’s correlation coefficient $$r = 0.3880, p\approx 4.06\times {10}^{-10}).$$ The length of an operation is also positively correlated with the ASA category (Spearman’s correlation coefficient $$r=0.1802$$, $$p=0.004$$; see Table 3). The length of operations tended to decrease with the number of operations carried out by the team (Spearman’s correlation coefficient $$r = -0.1591$$, $$p=0.01).$$

**Table 3:** Relationship between the length of an operation and ASA category

ASA category	Mean duration ± SD (min)
1	183.64 ± 47.23
2	195.99 ± 41.83
3	215.71 ± 49.88
4	222.50 ± 24.75

Operations on male patients were significantly longer than those on female patients (Welch’s *t*-test, $p = 0.0004$; see Table 4).

**Table 4:** Relationship between the length of an operation and a patient's sex

Sex	Mean duration ± SD (min)
Female	184.79 ± 39.03
Male	205.24 ± 44.76

The use of protective ileostomy is associated with longer operations (see Table 5, Welch’s *t*-test, $$p = 0.0005)$$. Importantly, ileostomy was used in most cases (232 cases, 92.8%).

**Table 5:** Relationship between the length of an operation and the use of protective ileostomy

Use of ileostomy	Mean duration ± SD (min)
No	158.00 ± 26.89
Yes	200.84 ± 43.81

### Multivariate regression analysis

The length of an operation depends not only on the experience of the surgical team and the methods used but also on the patient's physical characteristics. To account for all of these factors, we developed two multivariate regression models to estimate the expected length of an operation. In both models, the following factors related to the traits of the patients and the methods used were treated as potentially affecting the length of an operation: age, BMI, risk according to ASA, use of ileostomy, operating technique (0 = laparoscope, 1 = open), and frailty according to WHO. Due to the small frequencies of ASA categories 1 and 4, risk was defined as a binary variable with 0 corresponding to categories 1 and 2 and 1 corresponding to categories 3 and 4. The principal surgeon was also taken to be a factor by using three binary variables, ${S}_{1}$, ${S}_{2}$, and ${S}_{4}$, that indicated whether Surgeon 1, Surgeon 2, or Surgeon 4, respectively, led an operation. Surgeon 3 was used as a baseline for comparison, as this surgeon had led the most operations.

In the first model, the number of the operation in the sequence of all the operations performed by the team was used as a variable describing the team's experience. Finally, the number of the operation in the sequence of operations led by the current lead surgeon was used as a variable describing his/her experience.

Importantly, it is expected that a surgeon (or the team) learns more rapidly during the first operations than during later ones. Therefore, a second model was developed in which the number of the operation in the sequence was split into ten groups (operations 1–25, 26–50, 51–75, 76–100, 101–125, 126–150, 151–175, 176–200, 201–225, and 226–250). The operations numbered 226–250 were treated as the baseline, since it was expected that after an initial period, the mean length of an operation would stabilize. Hence, the number of the operation was coded using nine binary variables. Similarly, the number of the operation in the sequence of operations led by the current lead surgeon was split into four categories (operations 1–25, 26–50, 51–75, and 76 +). In this case, the operations numbered 1–25 were used as the baseline for comparison, since this was the category with the most observations.

In both cases, a regression model was developed using the method of stepwise elimination. Initially, all descriptive variables were included in the model. In each step, the variable associated with the largest *p*-value was removed from the model, until each of the remaining variables was significant at the 5% level. The final model was chosen by minimizing Akaike’s information score (see Akaike [8]). This score considers both the number of variables in a model and how well the model fits the data. Hence, simple models that fit the data well are favored.

Model 1 for the mean duration of an operation is given by the following equation:

$$T=98.18+16.75{X}_{1}+3.69{X}_{2}+30.92{X}_{3}-0.62{X}_{4}-28.02{X}_{5}-32.93{X}_{6}-41.37{X}_{7},$$

where ${X}_{1} \text{is }1$ when the patient is male and 0 when the patient is female, ${X}_{2}$ is the body mass index, ${X}_{3} \text{is }1$, when protective ileostomy is used, and otherwise 0, ${X}_{4}$ is the number of an operation in the sequence of operations carried out by the appropriate lead surgeon, ${X}_{5} \text{is }1$, when the lead operator is Surgeon 2, and otherwise 0, ${X}_{6} \text{is }1$ when the lead operator is Surgeon 4, and otherwise 0, ${X}_{7} \text{is }1$ when open surgery is used and 0 when a laparoscopic approach. The Akaike information score for this model is 2456.2.

Based on this model:

1. Operations on males take on average 16.75 min longer than those on females
2. The mean length of operations increases by 3.69 min per BMI unit
3. Use of protective ileostomy increases the length of the operation by just over half an hour on average
4. The mean length of an operation decreases with the number of the operation in the sequence of operations carried out by the appropriate principal surgeon (about 37 s/operation, i.e., after 100 operations by around an hour). This parameter relates to all four principal surgeons
5. The baseline (initial) speed of Surgeons 2 and 4 is around 30 min faster than that Surgeon 3. Notably, Surgeon 3 had already carried out > 30 operations when Surgeon 2 began leading operations and > 40 operations when Surgeon 4 began leading operations. Hence, considering the greater experience gained by Surgeon 3, there was no significant difference in the speeds of these operators over the period when all four surgeons were leading operations
6. There was no significant difference in the speeds of Surgeon 1 and of Surgeon 3.
7. Use of the open technique decreases the length of the operation by just over 40 min on average compared to use of the laparoscope

Model 2 for the mean duration of an operation is given by the following equation:

$$T=93.83+13.20{X}_{1}+3.71{X}_{2}+25.20{X}_{3}-31.98{X}_{6} -41.47{X}_{7}+10.63{X}_{8}-21.24{X}_{9}-17.72{X}_{10}-41.03{X}_{11}-45.09{X}_{12},$$

where ${X}_{1},{X}_{2}, {X}_{3}, {X}_{6} \text{and }{X}_{7}$ are defined as in Model 1; ${X}_{8}$ is 1 if Surgeon 1 was the principal surgeon, and otherwise 0; ${X}_{9}$ is 1 if the number of the operation in the sequence of all operations is between 76 and 100, and otherwise 0; ${X}_{10}$ is 1 if the number of the operation in the sequence of all operations is between 101 and 125, and otherwise 0; ${X}_{11}$ is 1 if the principal surgeon has led between 51 and 75 operations, and otherwise 0; ${X}_{12}$ is 1 if the principal surgeon has led > 75 operations and otherwise 0. The Akaike information score for this model is 2440.3. Hence, this model fits the data better than Model 1 (the increased complexity of the model results in a clear improvement in how the model fits the data).

**Fig. 1:**
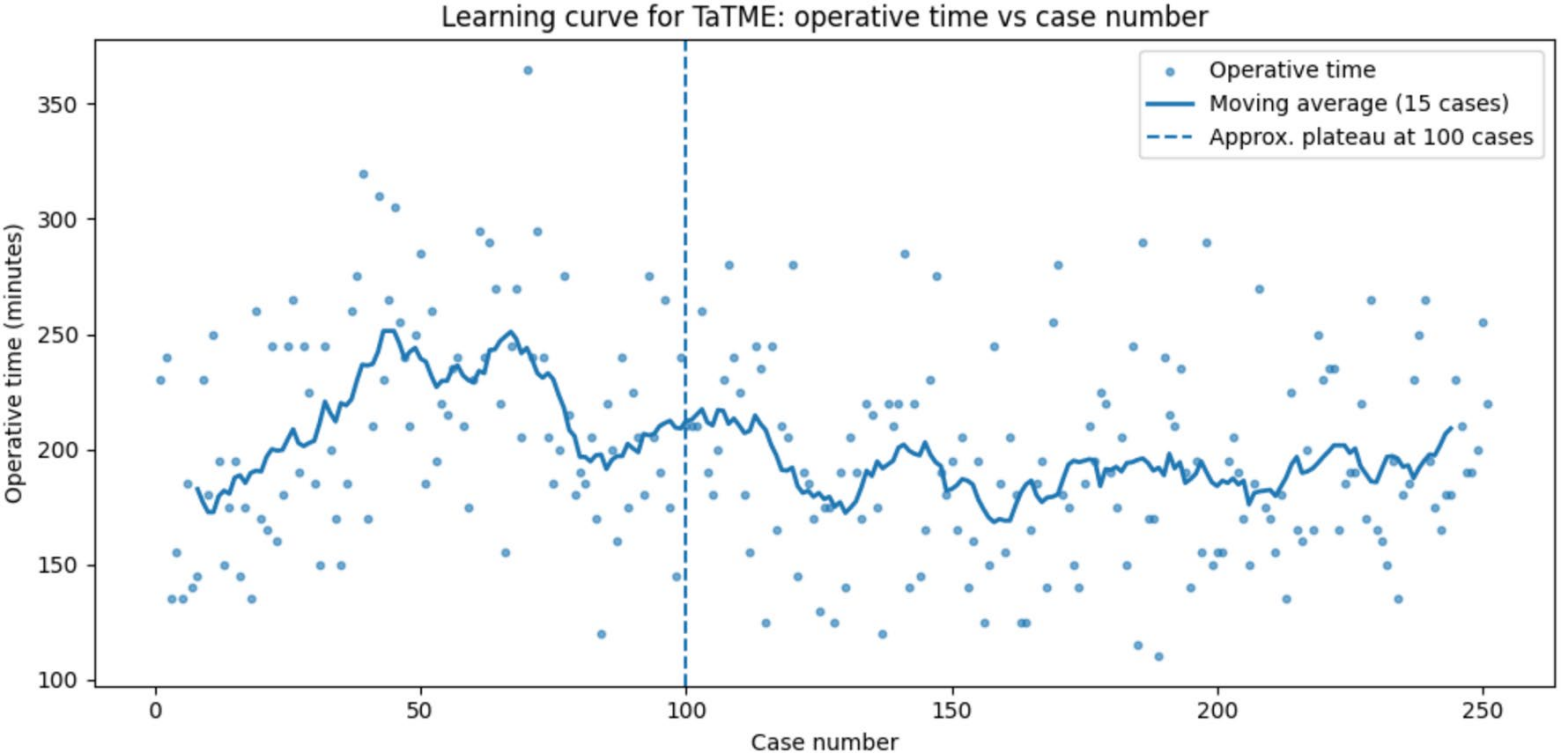
Learning curve for TaTME showing operative time by case sequence (dots) and a 15-case moving average (blue line). The dashed vertical line marks the plateau point at approximately 100 cases

Based on this model:

1. Operations on males take on average 13.20 min longer than those on females
2. The mean length of operations increases by 3.71 min per BMI unit. It can be concluded from (1) and (2) that the mean length of an operation increases with patient size
3. Use of ileostomy increases the length of the operation by just over 25 min on average
4. The initial speed of Surgeon 4 is about 32 min faster on average than that of Surgeon 3 [see point (7) for a more extensive description of the association between the mean length of operations and the principal surgeon]
5. Use of the open technique decreases the length of the operation by just over 40 min on average
6. The initial speed of Surgeon 1 is on average about 10 min slower than that of Surgeon 3. Since these two surgeons began leading operations at the beginning of the study period and led a similar number of operations, it can be concluded that Surgeon 3 carries out operations on average 10 min faster than Surgeon 1
7. The average duration of operations decreased progressively over time. In our cohort, cases 76–125 were approximately 20 min shorter than the first 75 procedures, while cases beyond 125 were about 40 min shorter. This pattern reflects the accumulation of collective team experience, with Surgeons 1 and 3 having already performed around 40–50 operations at this stage, resulting in reductions of approximately 40 min compared with their initial baseline. Surgeon 4 demonstrated operative times comparable to those of the most experienced operators from the early phase of their learning curve. The analysis indicates that operative time stabilized after approximately 100 cases for the team as a whole, corresponding to about 40–50 procedures per individual surgeon. The learning curve for TaTME is illustrated in Fig. 1, which plots operative time against sequential case number (*n* = 250) with a 15-case moving average smoothing line. Operative duration decreased markedly during the first 80–100 procedures, followed by a clear plateau after approximately the 100th case, with times stabilizing at around 190–200 min. This visual trend supports the regression analyses and confirms that the team’s learning curve for TaTME reached stabilization after roughly 100 cases.

Although univariate analysis initially suggested that open surgery was faster, this effect was driven by the fact that open procedures were concentrated in the earliest phase of the series. When operative technique (open vs. laparoscopic) was analyzed independently of the team’s experience, no significant association with operative time was observed (Welch's *t*-test, *p* = 0.44). By the end of the study period, laparoscopic operations were being completed in approximately the same time as open procedures performed at the beginning of the learning curve.

### Comparison of operative techniques

To provide a clearer picture of technique usage and operative duration, Table 6 summarizes the number of procedures performed using open, laparoscopic, and conversion techniques and their mean operative time and standard deviation. Overall, 208 procedures were completed laparoscopically (mean 201.3 min, SD 45.1 min), 37 were completed using an open abdominal phase (mean 196.8 min, SD 48.8 min), and 5 were laparoscopic‑to‑open conversions (mean 219.3 min, SD 30.5 min). These conversions often reflected complex cases. Although open procedures appeared faster in the unadjusted analysis, this difference was largely due to the fact that open surgery was used almost exclusively in the early part of the series, when the team had limited laparoscopic capacity.

**Table 6:** Comparison of operative time by surgical technique

Technique	Number of cases	Mean operative time ± SD (min)	Median (min)
Laparoscopic	208	201.3 ± 45.1	195
Open	37	196.8 ± 48.8	185
Conversion	5	219.3 ± 30.5	225

To summarize, the mean length of an operation increases with patient BMI. In the first half of the study period, almost all operations were led by Surgeon 1 or Surgeon 3. During this period, Surgeons 2 and 4 gained experience as team members and began leading operations. By the end of this initial period, Surgeons 1 and 3 were completing operations around 20 min faster on average than at the beginning of the study period. At this time, Surgeons 2 and 4 were completing operations at a similar pace. Soon after these two new principal surgeons began leading operations, the mean operation time stabilized at about 40 min faster than at the beginning of the study period (all other things being equal). In practice, the average operation time decreased by < 40 min over the study period, since the surgical team was carrying out more complex operations on average than at the beginning of the study period (e.g., laparoscopic rather than open operations).

### Oncological and clinical outcomes

In addition to operative time, we reviewed key short-term oncological and clinical outcomes. Positive circumferential resection margins were recorded in 3 of 250 cases (1.2%), and anastomotic leaks occurred in 27 of 250 patients (10.8%). Overall, 61 patients (24%) experienced postoperative complications according to the Clavien-Dindo classification, while 189 (76%) had an uneventful recovery. Of these complications, 24 cases (≈10%) were classified as ≥ Clavien-Dindo grade III, indicating major morbidity. There was no clear temporal trend in these outcomes across the learning phases. These rates are comparable to those reported in contemporary TaTME series and suggest that procedural refinement did not compromise oncological safety.

The completeness of mesorectal excision was assessed according to the MERCURY classification in cases with detailed pathological reporting, reflecting the gradual implementation of this standardized assessment at our institution. Among these evaluated specimens, complete mesorectal excision was achieved in 107 cases (77.0%), nearly complete in 25 (18.0%), and incomplete in 7 (5.0%). The proportion of complete specimens remained stable throughout the study period, indicating consistent oncological quality despite reductions in operative time.

A more detailed analysis of these oncological and clinical outcomes will be presented in forthcoming dedicated publications.

## Discussion

All procedures in our series were executed using a synchronous two‑team strategy, with an abdominal and a transanal team working concurrently. During the earliest phase, an open abdominal approach was used because of a shortage of laparoscopic surgeons, after expanding the team, we transitioned to a fully minimally invasive abdominal phase. Only a few open procedures were performed in later years, owing to logistical or patient‑specific complexities. This operational framework likely facilitated the efficiency gains observed during the learning curve.

The findings of this study suggest the presence of a substantial learning curve in the implementation of TaTME. According to our regression models, a significant reduction in operative time was observed after approximately 100 cases performed by the leading surgeons, consistent with previously published data suggesting that at least 40–50 procedures are required to achieve technical proficiency [4, 5]. This is particularly important in the context of safe implementation strategies and structured training programs [9, 10]. These results are consistent with conclusions from other studies supporting the use of structured, proctored TaTME training pathways, such as the one proposed by the international TaTME consensus group, which emphasizes simulation-based training, dual-team practice, and mentoring [9, 10].

Our analysis revealed that the mean duration of TaTME procedures across the entire cohort was 200.52 min. This aligns with durations reported in major multicenter trials such as the COLOR III trial (~ 210 min) [3] and the GRECCAR 11 study (~ 250 min in the TaTME arm) [6], though with some variance likely due to differences in patient selection, team experience, and institutional protocols. In Penna et al.'s international registry analysis of 720 TaTME cases, the median operative time was approximately 270 min, further underlining the variability between centers [5].

**Fig. 2:**
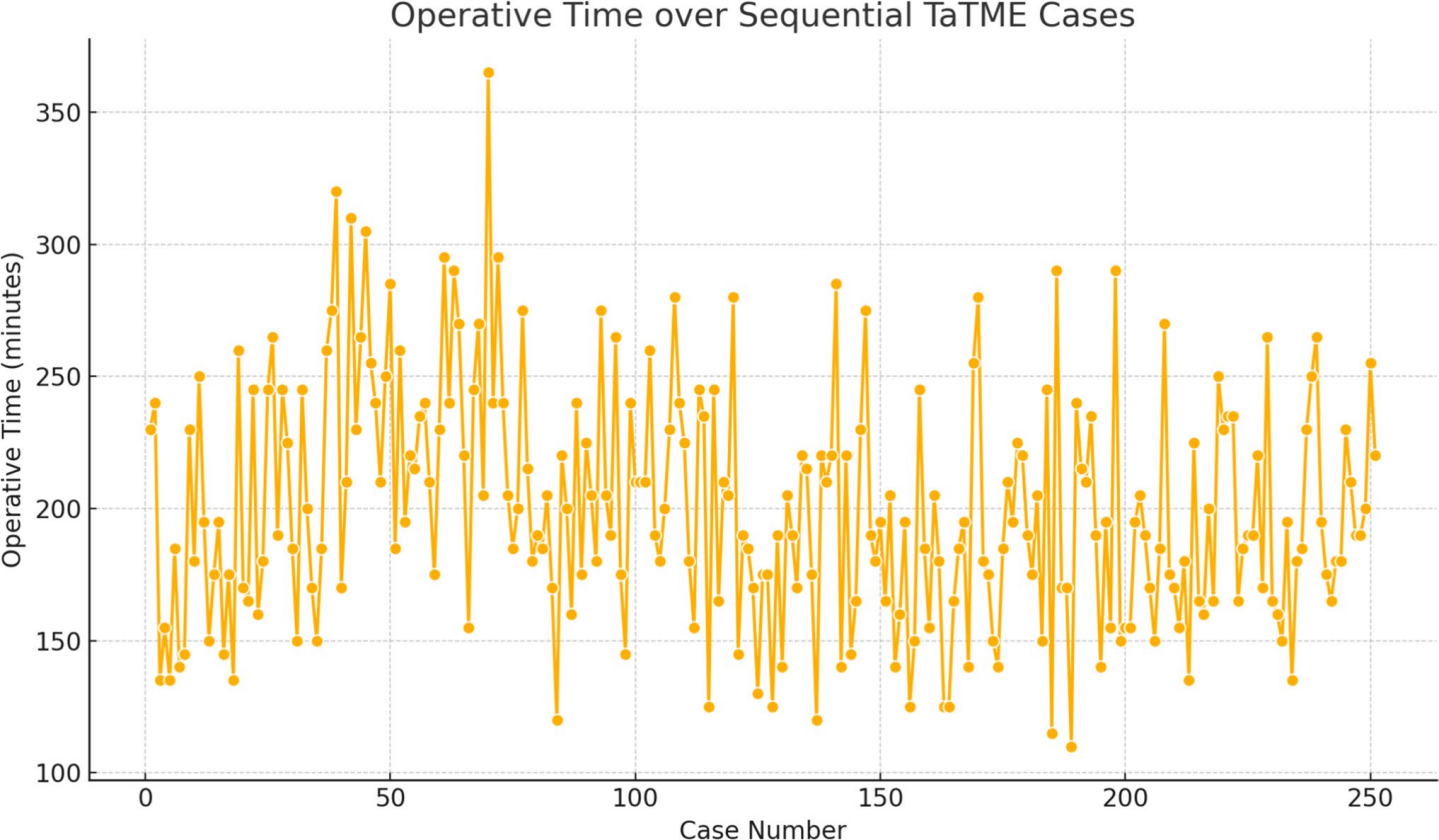
Operative time over case sequence (learning curve)

A notable finding in our dataset was that operative times decreased progressively with increasing experience, at the levels of both individual surgeons and the overall team. Our multivariate models estimate a reduction of approximately 40 min over the study period, correlating with the learning curve phase. This trend is illustrated in Fig. 2, which demonstrates the gradual decline in operative time across successive cases. Given the variability in outcomes during the learning phase, several national programs now advocate for prospective outcome auditing during the first 50–100 TaTME cases per institution [11].

**Fig. 3:**
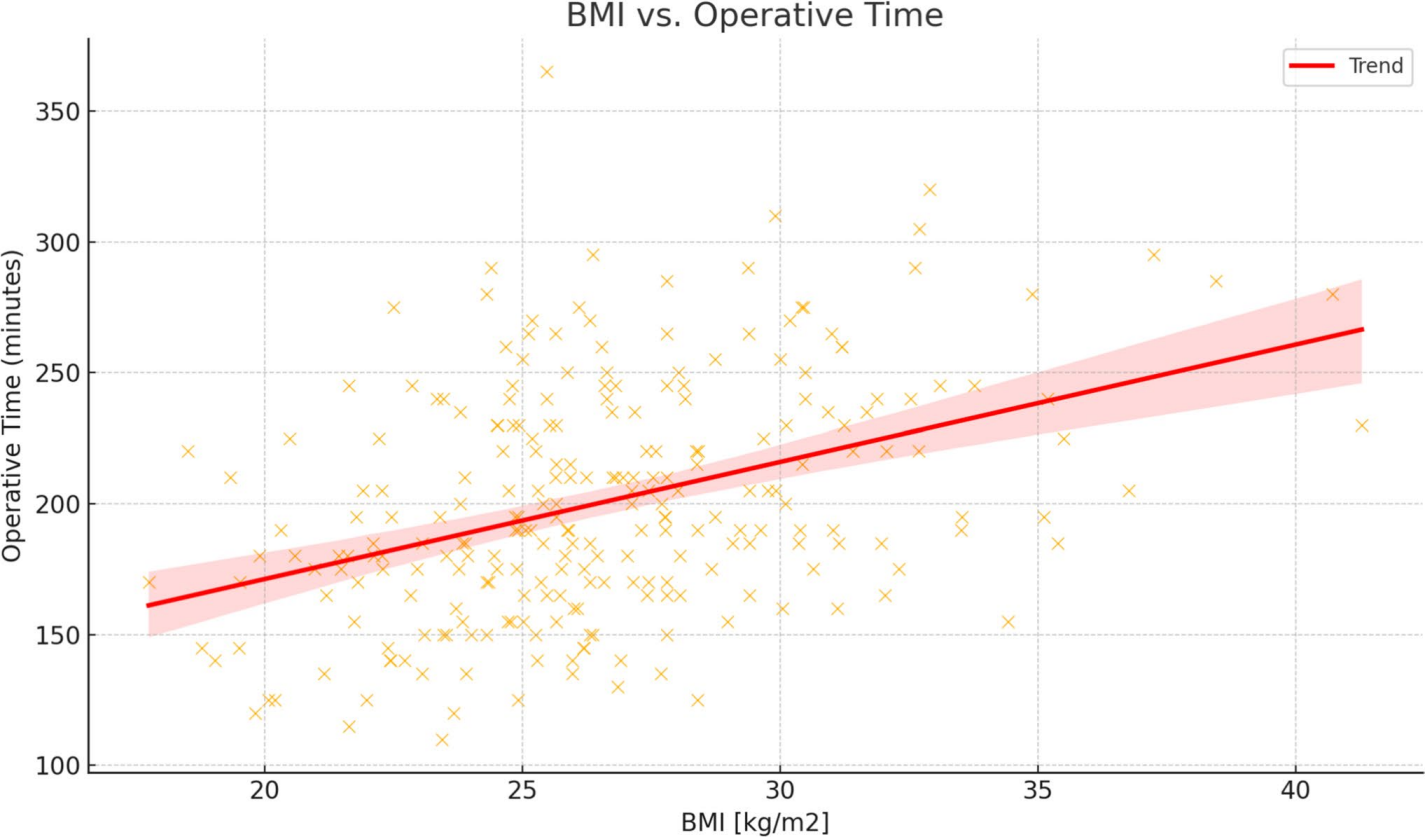
Correlation between BMI and operative time

Patient-related factors also significantly influenced operative time. Male sex was associated with longer procedures, by an average of ~ 13–16 min, in line with anatomical challenges commonly encountered in the narrow male pelvis. This observation is consistent with previous studies indicating increased technical difficulty in male patients undergoing rectal surgery [12, 13]. BMI was positively correlated with operative time, with each unit increase in BMI extending the procedure by approximately 3.7 min. These findings align with prior analyses suggesting that obesity increases the technical difficulty, particularly in minimally invasive pelvic dissection [14–16]. The strength of this correlation is shown in Fig. 3. Furthermore, patients classified as frail or with higher ASA categories (3–4) experienced longer procedures, suggesting that comorbidities may also affect intraoperative decision-making and tempo.

The technical approach, specifically the use of a protective ileostomy, was also associated with significantly longer surgeries. This is understandable, given the additional steps required for stoma creation and the intraoperative judgment often involved in high-risk anastomosis cases.

Interestingly, while open surgery appeared to be faster in unadjusted comparisons (by ~ 40 min), this difference disappeared after adjusting for surgeon experience and patient complexity. This reflects the maturation of laparoscopic skills in our institution and suggests that with appropriate training and experience, minimally invasive approaches can achieve comparable efficiency to open techniques. Some centers have suggested that robotic TME may offer similar pelvic access benefits with a potentially shorter learning curve, although current comparative data remain inconclusive [17].

**Fig. 4:**
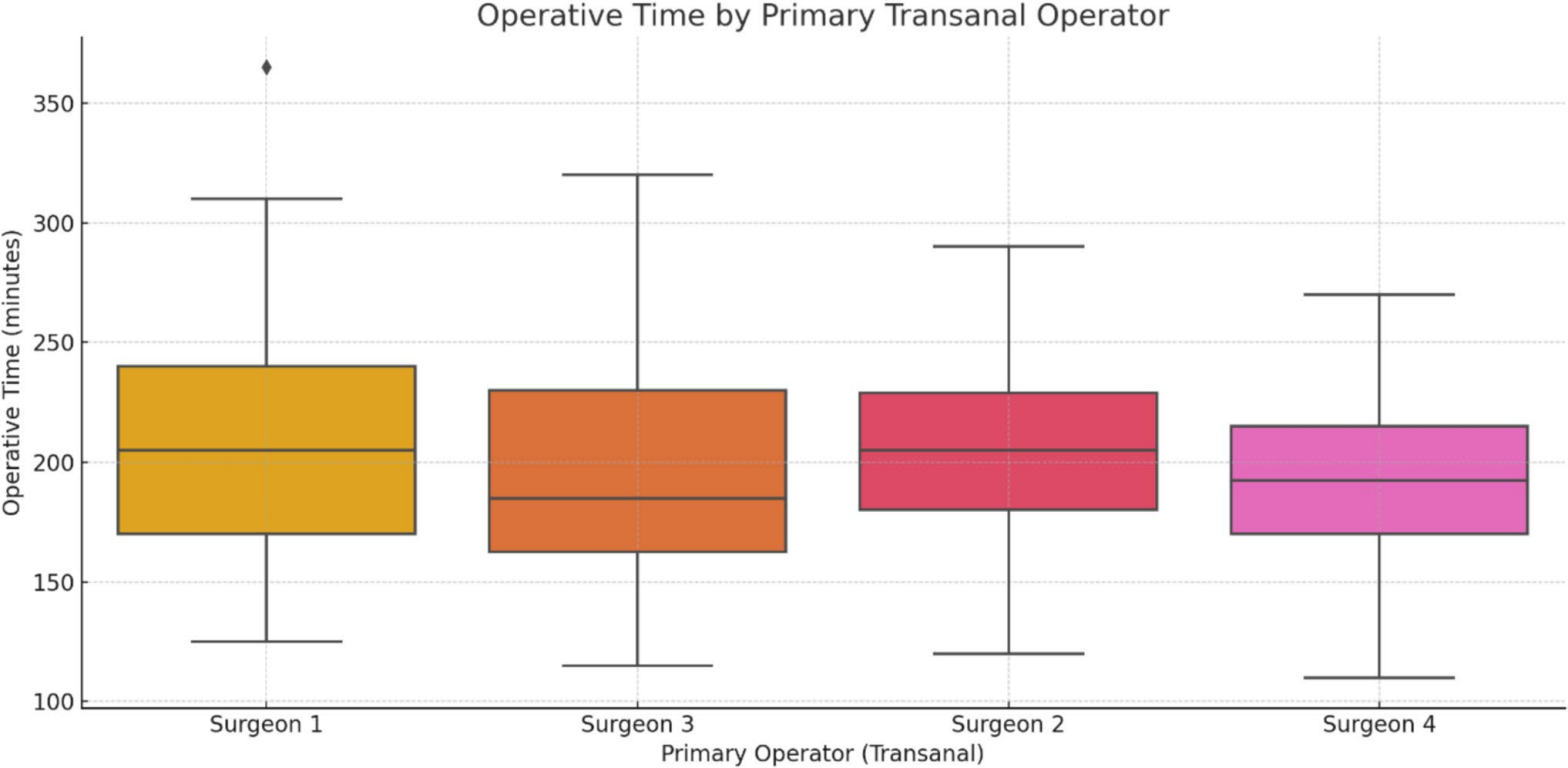
Operative time by primary transanal surgeon

A comparison of lead transanal surgeons revealed variation in operative times; Fig. 4 presents this distribution across the four anonymized surgeons. These differences underline the role of individual learning trajectories, even within standardized protocols.

These observations highlight the importance of experience-based credentialing and suggest that operative duration can be used as a quality indicator during the learning phase. The results also underscore the need for individualized operative planning that accounts for patient sex, BMI, and ASA classification to optimize scheduling and resource allocation.

These findings align with broader perspectives in the field, as highlighted in the comprehensive review by Vignali et al., which emphasized the need for careful patient selection, surgical expertise, and long-term data to fully establish the role of TaTME in rectal cancer treatment [18].

## Limitations

This study is subject to the limitations of its retrospective design and single‑center setting. The distribution of open versus laparoscopic cases was heavily skewed toward the early phase, potentially introducing residual confounding despite multivariable adjustment. Our results reflect the performance of a specialized, high‑volume team using a two‑team approach and may not generalize to smaller centers or single‑surgeon practice. Functional outcomes such as continence and low anterior resection syndrome were not analyzed, as these are the subject of separate ongoing studies.

## Conclusion

Our study suggests that the duration of TaTME procedures decreases significantly with accumulating experience, at both the individual surgeon and surgical team levels. The learning curve appears to stabilize after approximately 100 cases. Male sex, higher BMI, and increased ASA score are associated with longer operative times, and their consideration is essential during preoperative planning. While open surgery initially appears faster, the gap closes as laparoscopic experience increases, supporting the feasibility of a fully minimally invasive approach in experienced hands. These findings may help inform future training guidelines, procedural credentialing, and workflow optimization in centers implementing TaTME.

## Data Availability

The datasets generated and/or analyzed during the current study are available from the corresponding author on reasonable request.
